# Supplementary material for: Variable PD-1 glycosylation modulates the activity of immune checkpoint inhibitors
Source: Life Sci Alliance. 2024 Jan 4;7(3):e202302368. doi: 10.26508/lsa.202302368 (PMC10766783; doi:10.26508/lsa.202302368)
Supplement: Supplementary file 1 [file LSA-2023-02368_TableS1.docx]

**Supplemental Table 1.** Therapeutic anti-PD-1 antibodies approved or in review in the EU or US. Data adapted from *Antibody therapeutics approved or in regulatory review in the EU or US*. The Antibody Society (2023, Nov, 22)

| **International non- proprietary name, US product identifier** | **Brand name** | **Target;**  **Format** | **Indication first approved or reviewed** | **First EU approval year** | **First US approval year** | **Estimated PDUFA date or FDA action** |
| --- | --- | --- | --- | --- | --- | --- |
| Nivolumab | Opdivo | PD1; Human IgG4 | Melanoma, non-small cell lung cancer | 2015 | 2014 |  |
| Pembrolizumab | Keytruda | PD1; Humanized IgG4 | Melanoma | 2015 | 2014 |  |
| Cemiplimab, cemiplimab-rwlc | Libtayo | PD-1; Human mAb IgG4 | Cutaneous squamous cell carcinoma | 2019 | 2018 |  |
| Dostarlimab, dostarlimab-gxly | Jemperli | PD-1; Humanized IgG4 | Endometrial cancer | 2021 | 2021 |  |
| Retifanlimab, retifanlimab-Dlwr | Zynyz™ | PD-1; Humanized IgG4 | Merkel cell carcinoma | In review | 2023 |  |
| Serplulimab | HANSIZHUANG | PD-1; Humanized IgG4 | Small cell lung cancer | In review | NA |  |
| Camrelizumab | AiRuiKa | PD-1; Humanized IgG4 | Hepatocellular carcinoma | NA | In review |  |
| Toripalimab | Tuoyi | PD-1; Humanized IgG4 | Nasopharyngeal carcinoma | In review | 2023 |  |
| Penpulimab | (Pending) | PD-1; Humanized IgG1 | Metastatic nasopharyngeal carcinoma | NA | In review | Status unknown as of Jan 2023; Real-Time Oncology Review |
| Sintilimab | (Pending) | PD-1; Human IgG4 | Non-small cell lung cancer | NA | 2nd cycle review | CR letter issued Mar 2022 |
| Tislelizumab | (Pending) | PD-1; Humanized IgG4 | Esophageal squamous cell carcinoma | 2023 | 2nd cycle review | CR letter issued in Mar 2022 |
