# Supplementary material for: Variable PD-1 glycosylation modulates the activity of immune checkpoint inhibitors
Source: Life Sci Alliance. 2024 Jan 4;7(3):e202302368. doi: 10.26508/lsa.202302368 (PMC10766783; doi:10.26508/lsa.202302368)
Supplement: Supplementary file 2 [file LSA-2023-02368_TableS2.docx]

**Supplemental Table 2.** Study population characteristics. Counts are followed by the appropriate column-wise percentage, while continuous variables are summarized by medians and standard deviation.

| **Characteristics** | **iSpecimen**  (N=21) |
| --- | --- |
| Age | 65.3 (7.9) |
| BMI | 27.6 (4.0) |
| Male | 13 (61.9%) |
| Race |  |
| Caucasian | 21 (100%) |
| Disease Category |  |
| NSCLC Stage I | 6 (28.6%) |
| NSCLC Stage II | 5 (23.8%) |
| NSCLC Stage III | 7 (33.3%) |
| NSCLC Stage IV | 3 (14.3%) |
| Histology |  |
| Adenocarcinoma | 8 (38.1%) |
| Bronchioloalveolar carcinoma | 1 (4.8%) |
| Squamous cell carcinoma | 12 (57.1%) |
| Smoking History |  |
| Non-smokers | 7 (33.3%) |
| Former smokers | 1 (4.8%) |
| Smokers | 13 (61.9%) |
